# Metabolic and Functional Connectivity Changes in Mal de Debarquement Syndrome

**DOI:** 10.1371/journal.pone.0049560

**Published:** 2012-11-29

**Authors:** Yoon-Hee Cha, Shruthi Chakrapani, Alexis Craig, Robert W. Baloh

**Affiliations:** 1 Department of Neurology, University of California Los Angeles, Los Angeles, California, United States of America; 2 Semel/Resnick Institute, University of California Los Angeles, Los Angeles, California, United States of America; 3 Cognitive Sciences, University of California Irvine, Irvine, California, United States of America; University of Iowa, United States of America

## Abstract

**Background:**

Individuals with *mal de debarquement syndrome* (MdDS) experience a chronic illusion of self-motion triggered by prolonged exposure to passive motion, such as from sea or air travel. The experience is one of rocking dizziness similar to when the individual was originally on the motion trigger such as a boat or airplane. MdDS represents a prolonged version of a normal phenomenon familiar to most individuals but which persists for months or years in others. It represents a natural example of the neuroplasticity of motion adaptation. However, the localization of where that motion adaptation occurs is unknown. Our goal was to localize metabolic and functional connectivity changes associated with persistent MdDS.

**Methods:**

Twenty subjects with MdDS lasting a median duration of 17.5 months were compared to 20 normal controls with ^18^F FDG PET and resting state fMRI. Resting state metabolism and functional connectivity were calculated using age, grey matter volume, and mood and anxiety scores as nuisance covariates.

**Results:**

MdDS subjects showed increased metabolism in the left entorhinal cortex and amygdala (z>3.3). Areas of relative hypometabolism included the left superior medial gyrus, left middle frontal gyrus, right amygdala, right insula, and clusters in the left superior, middle, and inferior temporal gyri. MdDS subjects showed increased connectivity between the entorhinal cortex/amygdala cluster and posterior visual and vestibular processing areas including middle temporal gyrus, motion sensitive area MT/V5, superior parietal lobule, and primary visual cortex, while showing decreased connectivity to multiple prefrontal areas.

**Conclusion:**

These data show an association between resting state metabolic activity and functional connectivity between the entorhinal cortex and amygdala in a human disorder of abnormal motion perception. We propose a model for how these biological substrates can allow a limited period of motion exposure to lead to chronic perceptions of self-motion.

## Introduction

In an 1881 article in the Lancet, J.A. Irwin wrote about a common phenomenon that occurs after sea travel: “This leads to the question of how all the phenomena of seasickness have usually a rapid tendency to pass away … the new habit may become so strong that a disturbance of it, by a return to the land, will be marked by similar phenomena; hence the unsteady gait sometimes observable…after a long and stormy voyage [1].” The unsteadiness that occurs after one disembarks from a boat or plane is a common phenomenon that normally ceases within two days of returning to land [2], [3]. However, in some individuals, a sensation that they are still rocking on the boat persists for months or years leading to imbalance, visuospatial problems, and cognitive dysfunction [4]. When this phenomenon becomes persistent, it is formally known as *mal de debarquement syndrome*, literally meaning “the sickness of disembarkment” (MdDS) [5].

Very little is known about the underlying neurobiology that leads to persistent MdDS as there are no scientific studies that have established the underlying anatomical cause of this perceptual disorder.

Case series have shown that there are no inner ear or structural brain abnormalities associated with MdDS, which is not unexpected since the triggers that lead to persistent MdDS such as going on a cruise, flying in an airplane, or driving for long distances are unlikely to cause structural brain or inner ear damage [4]. However, MdDS may be a disorder of neuroplasticity in which entrainment to a periodically moving environment changes the functional state of the brain making it difficult to readapt to stable land conditions. Individuals with MdDS feel much better when back in motion again, such as getting back on the boat or driving in a car [4], [6]. The inability to shut off the mechanisms that become activated during motion exposure that prevent motion sickness (getting one's “sea-legs”) may be related to aging and hormonal factors, as MdDS is typically a disorder of middle-aged individuals and is more common in women [7].

The localization for this persistent motion illusion has not been previously sought with functional neuroimaging though previous theories have included “vestibular flashbacks,” and “internal modeling” of the external motion suggesting a central localization [6], [8]. The localization of a key hub of motion adaptation, one that can remain persistently active after the stimulus is removed, would represent a remarkable form of neuroplasticity that is relevant to brain adaptability in aging and that also plays a role in motion intolerance.

We hypothesized that the persistent motion illusion of MdDS would be reflected by changes in brain metabolism and functional connectivity involving areas that process and store spatial information. MdDS represents a natural experimental model of the neuroplasticity of the motion adaption system and though it is an uncommon cause of imbalance, it may inform a deeper understanding of how motion information is stored in the brain.

## Methods

### Ethics Statement

Study procedures were completed according to Declaration of Helsinki guidelines and were approved by the Institutional Review Board of the University of California Los Angeles. The study was specifically approved by Medical IRB 3 which oversees neuroscience research. Subjects provided written informed consent.

Patients with a history of persistent MdDS meeting the following criteria were recruited through a University Neurology clinic: 1) A chronic perception of rocking dizziness that started after passive motion exposure such as from sea, air, or land travel; 2) Symptoms lasted at least one month; 3) Normal inner ear function testing with video or electronystagmography and audiograms; 4) Normal structural brain imaging with a non-contrast brain MRI; 5) First lifetime episode of MdDS; 6) No other cause for symptoms after evaluation by a neurologist.

Controls who did not have a history of dizziness or balance problems were recruited through advertisements and were screened for dizziness and neurological disorders. All subjects completed the Edinburgh handedness inventory [9] and the Hospital Anxiety and Depression Scale (HADS) [10].

### PET images

Subjects fasted for >2 hours prior to undergoing the PET scan. No subject had a finger stick glucose of >140 mg/dL. After injection of 5 mCi (185 MBq) of ^18^F-FDG into an antecubital vein, subjects sat with their eyes closed in a quiet dark room for the 40-minute uptake period. They were then scanned in 3D acquisition mode with a 30-minute emission scan followed by a 20-minute transmissions scan on a Siemens ECAT EXACT HR+ Scanner. Sixty-three slices were obtained with an axial resolution of 2.4 mm. Images were corrected for scatter, decay, scanner dead time, and attenuation and were reconstructed by back-projection using an all pass ramp filter of 2 mm full width half-maximum (FWHM). Attenuation correction was performed using a ^68^Germanium source. The preprocessed images obtained had a resolution of 128×128 voxels with a zoom of 2.75 mm.

### MRI images

Structural scans. Subjects were scanned on a Siemens Magnetom Trio 3Tesla scanner with a 12-channel head coil. Specifications for structural images were as follows: 192 slices at 1 mm slice thickness, voxel size: 1.0 mm×1.0 mm×1.0 mm, FoV: 256 mm, flip angle: 9 degrees, TR = 1900 ms, TE = 3.25 ms.

#### Resting state scans

The fMRI scanning parameters for resting state BOLD data acquisition were as follows: Whole-brain gradient-echo planar imaging sequences of 36 slices at 3 mm thickness, voxel size: 3.4 mm×3.4 mm×3.0 mm, FoV: 220 mm, TR = 2000 ms, TE = 30 ms, 120 volumes.

#### Frontal eye field (FEF) and motion sensitive area (V5/MT) localization

The fMRI scanning parameters for task based BOLD data acquisition were as follows: Whole-brain gradient-echo planar imaging sequences of 36 slices at 3 mm thickness, voxel size: 3.4 mm×3.4 mm×3.0 mm, FoV: 220 mm, TR = 3000 ms, TE = 30 ms; 96 volumes for the saccade task and 140 volumes for V5/MT localization.

To localize motion sensitive area V5/MT, subjects were shown a random dot kinematogram in which 21 seconds of random dot motion were alternated with 21 seconds of coherent motion moving in either the NW-SE or NE-SW direction. Subjects maintained fixation on a center fixation dot during stimulation and were shown 10 blocks of coherent motion alternating with 10 blocks of random motion.

For the saccade task, subjects were asked to look to each side of a black screen while a small white box flashed alternately from the right and left sides. Each stimulus was given for one second at a position 10 degrees from the center fixation point. Nine saccades to each side (18 seconds total) were followed by an 18 second resting block. Subjects were shown eight pairs of the saccade/rest blocks.

Eye tracking was performed with Restech ® video goggles using Arrington ViewPoint Eye Tracking® software to ensure that the subjects performed the appropriate tasks.

### PET image analysis

All image analyses for PET data were performed using Statistical Parametric Mapping (SPM) 8 (http://www.fil.ion.ucl.ac.uk/spm) with Matlab 2009b (Mathworks, Natick, MA.). The high-resolution structural images were realigned to the mean image, co-registered to the PET image and then normalized to the standard T1 template in spm8 in Montreal Neurological Institute (MNI) space. Images were smoothed with a 8 mm Gaussian kernel at FWHM. Grand-mean scaling, implicit masking and proportional normalization were performed. Voxels with a normalized intensity <0.8 were excluded in order to remove signal from cerebrospinal fluid and skull. A two-sample *t* test was performed between the smoothed normalized summed PET images of the MdDS vs. Control (CTRL) groups in whole brain analysis at z>3.3. Subjects' scores on the Hospital Anxiety and Depression Scale and age were used to regress out signal that co-varied with age and high depression and anxiety scores.

### MRI image analysis

#### Voxel based morphometry (VBM)

Structural images were reoriented using SPM5 to align the images in the anterior and posterior planes. Using the Matlab based VBM5 toolbox, each image was segmented into grey matter, white matter and CSF volumes in each subject's native brain space. Modulated images were created using medium hidden Markov random field weighting. After normalization, the images were smoothed with a 8 mm Gaussian kernel at FWHM and a sample homogeneity test was performed to remove any outliers. The Marsbar (http://marsbar.sourceforge.net) toolbox was used to extract GM values from ROIs defined by PET.

#### FEF, V5/MT, and V1 localization

Contrast images for “coherent motion-minus-random motion” at a whole brain corrected threshold of *z*>3.3 were created for each individual subject and the individual contrast images were averaged for the MdDS group and for the CTRL group separately. Similarly, the contrast images for “saccade-minus-fixation” were created for each individual and averaged for MdDS subjects and CTRL groups at a whole brain corrected threshold of *z*>3.3. Multiple regression analysis on all visual stimulation data was performed to show brain areas that had the highest correlation with visual stimulation, to define right and left areas V1.

#### Functional connectivity

Image analysis was performed using Data Processing Assistant for Resting-State fMRI (DPARSF), a MATLAB based toolbox [11]. The first 10 images were removed to account for equilibration, were realigned to the center image, and normalized to a standard T1 template. After spatial smoothing, global trends were removed and a map of the amplitude of low frequency fluctuation (ALFF) was created. Meantime series within specified ROIs were extracted from the ALFF maps.

Pearson's correlation coefficients were determined between ALFF maps at each of the peak voxels in each ROI and were converted by Fisher's r-to-z transformation into z scores (NOTE: the z scores on PET analysis are statistical z's, a measure of standard deviations from the mean. z scores for functional connectivity are Fisher's z's, a measure of correlation). Brain areas that have high correlations in spontaneous activity are functionally connected.

## Results

### Clinical History

Twenty subjects with MdDS (mean age: 43.4 years, SEM: 2.50, Range: 27–66 years) participated in the study. The MdDS subjects included 15 women and 5 men, with a median duration of symptoms lasting 17.5 months (range 3–240 months). Eleven subjects developed symptoms after sea travel (typically a cruise); eight after air travel, and one after a train ride. All but one subject was still symptomatic during a follow-up period of 12–36 months after they were imaged. These MdDS subjects were compared to 20 controls matched for age, sex, and handedness (mean age: 43.0 years, SEM: 2.91, Range 23–59 years). There were two-left-handed subjects in each group. Two sample independent Wilcoxon rank sum test for age was 414 for the MdDS group and 406 for CTRL, with *p* = 0.914. All subjects had normal neurological exams.

### PET analysis

**Figure 1:**
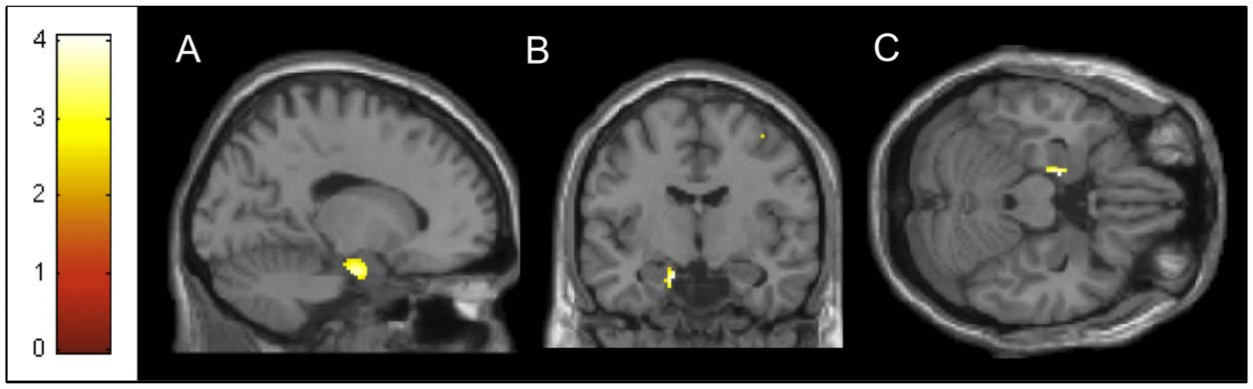
Area of relative hypermetabolism in MdDS subjects compared to controls. Cluster with peak voxel z>3.3 for the MdDS>CTRL contrast (MNI coordinates −14 −8 −22) centered at the entorhinal cortex/amygdala transition shown in the a) sagittal; b) coronal; c) axial planes. Image is presented at *z*>2.57 for better visualization; extent voxels: 0.

**Figure 2:**
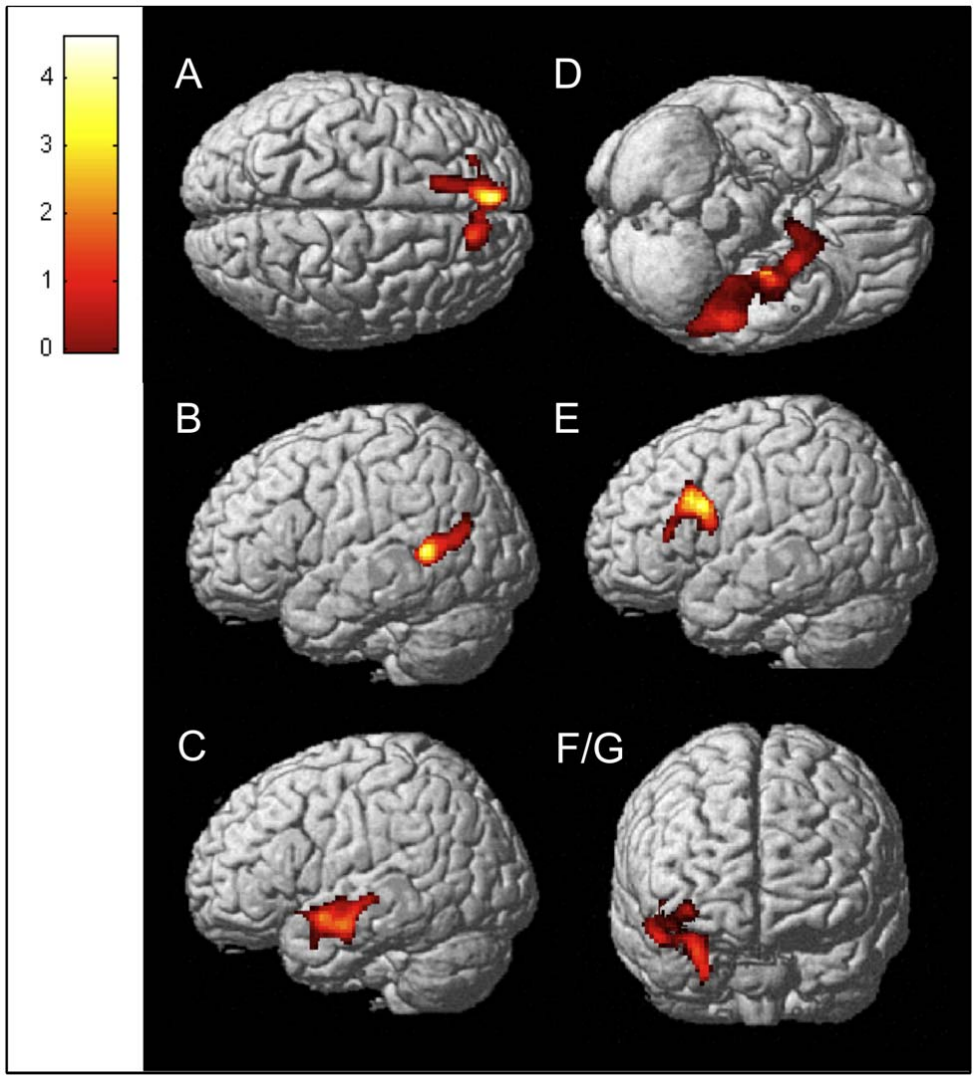
Areas of relative hypometabolism in MdDS subjects compared to controls. Clusters with peak voxel z>3.3 for the CTRL>MdDS contrast: a) Left superior medial gyrus; b) Left middle temporal gyrus; c) Left superior temporal gyrus; d) Left inferior temporal gyrus; e) Left middle frontal gyrus; f/g) Right amygdala and insula. Images are shown at *z*>1.96 for better visualization; extent voxels: 0.

One cluster in the MdDS>CTRL contrast at MNI coordinate x = −14; y = −8, z = −22 exceeded threshold at z>3.3 (**Figure 1**). The probability atlas of Jülich in the Anatomy toolbox maps the peak voxel to between the left entorhinal cortex and the superficial group (SF) of the amygdala [12] (**Table 1**). Cortical areas in the CTRL>MdDS contrast significant at z>3.3 included the left superior medial gyrus, left middle frontal gyrus left superior temporal gyrus, left middle temporal gyrus, left inferior temporal gyrus, right amygdala and right insula (these two were part of one large cluster) (**Figure 2** **,** **Table 1**).

**Table 1:** Peak voxels of significance in MdDS>CTRL and CTRL>MdDS contrasts showing unadjusted and adjusted z scores for grey matter volume.

Contrast	X	Y	Z	ke	z_unadj_	z_adjVBM_	Localization
MdDS>CTRL	−14	−8	−22	12	3.66	3.61	Left entorhinal cortex/amygdala
CTRL>MdDS	−8	52	36	39	4.03	4.01	Left superior medial gyrus
	−50	−52	8	40	3.86	3.25	Left middle temporal gyrus
	−52	0	−14	41	3.67	3.84	Left superior temporal gyrus
	−52	−38	−24	42	3.56	3.63	Left inferior temporal gyrus
	−32	18	30	38	3.53	2.05	Left middle frontal gyrus
	30	−2	−26	13	3.45	3.39	Right amygdala
	40	2	−8	13	3.31	3.34	Right insula

#### Correcting for grey matter probability

After correction for grey matter volume, all clusters remained significant at z>1.96, and all but the left middle frontal gyrus cluster remained significant at z> 3.3 (Table 1).

#### Ruling out correlation with depression and anxiety scores

The mean HADS anxiety subscore in the MdDS group was 8.8+/−4.7 versus 3.4+/−3.6 in controls (*p*<0.001). The mean depression subscore in the MdDS group was 6.5+/−5.2 versus 1.6+/−2.1 in controls (*p*<0.001). On the HADS rating scale, 0–7 = normal, 8–10 = borderline, and 11–21 = abnormal indicating borderline anxiety and high normal depression scores in the MdDS subjects. Although the primary analysis had used age and the HADS scores as nuisance covariates we also performed multiple regression analysis to show voxels that have the highest correlation with high anxiety or high depression scores using scores from all 40 subjects (20 MdDS+20 CTRL). Because depression and anxiety are often comorbid conditions, depression specific areas were obtained by the Depression>Anxiety contrast, and anxiety specific areas were obtained by the Anxiety>Depression contrast.

The resulting voxels did not show any overlap with any of the regions seen in the primary analysis, even at a more liberal threshold of *z*>1.96. This analysis did reveal other areas previously known to be highly relevant in mood disorders. The top 5 clusters that correlated with high depression and high anxiety scores are presented in **Table S1** and **Figures S1 and S2**. Of particular significance was the subgenual anterior cingulate cortex activity correlating with high depression scores and the dorsal midbrain and anterior temporal lobe activity correlating with high anxiety scores.

#### Functional connectivity

Because the entorhinal cortex has extensive connections with much of the neocortex, we determined whether there might be a relationship between the entorhinal cortex/amygdala (EC/AG) hypermetabolism and areas of relative hypometabolism. In addition, functional connectivity was calculated between the EC/AG focus and the ipsilateral FEF, V5/MT, superior parietal lobule (SPL), and V1 in order to determine whether there is an underlying difference in the pattern of connectivity between the EC/AG and anterior versus posterior neocortex. The superior parietal lobule, a key area of spatial information processing was anatomically defined in the Jülich atlas. All others were functionally defined.

**Figure 3:**
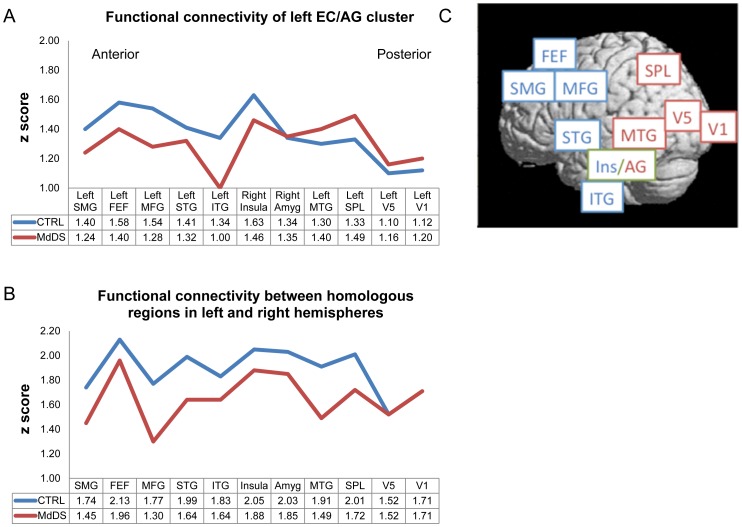
Functional connectivity differences in subjects with MdDS compared to controls. a) Functional connectivity between the left EC/AG hypermetabolic focus in MdDS and regions of relative hypometabolism, shown in the context of landmark areas FEF, SPL, V5/MT, and V1. b) Functional connectivity between homologous brain regions. c) Foci are presented anterior to posterior anatomically. Blue line: Controls; Red line: MdDS.

**Figure 3a** shows decreased functional connectivity between the EC/AG and prefrontal, premotor, superior temporal, and inferior temporal foci in MdDS subjects compared to CTRLs, whereas connectivity is increased between EC/AG and middle temporal, SPL, V5/MT, and V1, all areas that process either visual or vestibular information. Connectivity to contralateral insula was also reduced to a lesser degree and connectivity to the contralateral amygdala was comparable to CTRLs.

In order to determine whether functional connectivity changes between EC/AG and other foci were reflective of *general* connectivity differences between MdDS subjects and controls rather than specific connectivity changes related to the EC/AG focus, connectivity measurements were made between homologous brain regions starting from bilateral FEFs moving posteriorly to bilateral areas V1 (**Figure 3b**). **Figure 3c** shows the relative locations of each of these loci. Functional connectivity between homologous brain regions extending from the FEFs all the way to SPL was reduced in MdDS subjects whereas it was exactly the same between bilateral areas V5/MT and V1 compared to CTRLs. Connectivity between EC/AG and the ipsilateral middle temporal gyrus and SPL were increased despite overall reduced connectivity between hemispheres at these levels. Thus, functional connectivity between these posterior temporal and occipital areas and area EC/AG was increased relative to the general pattern of connectivity differences seen between MdDS and CTRL subjects.

## Discussion

The neural substrates for persistent MdDS have never been previously investigated with functional neuroimaging and this study presents an anatomical basis for what has been previously considered to be a completely subjective syndrome of chronic rocking dizziness. We show that MdDS, a disorder of continuous self-motion perception is associated with hypermetabolism of the left EC/AG, decreased prefrontal and temporal lobe metabolism, increased functional connectivity with posterior spatial processing areas, and decreased connectivity with several prefrontal areas. Functional connectivity was reduced between all homologous regions in frontal, temporal, and parietal lobes in MdDS subjects while being preserved for posterior temporal (V5/MT) and primary visual cortex (V1).

These data implicate the entorhinal cortex and amygdala in a human disorder of persistent motion perception and expand the role that EC/AG plays in human spatial memory. This is the first study to show metabolic abnormalities in patients with a chronic illusion of motion and provides information that may expand the relevance of limbic function to motion perception.

The entorhinal cortex is situated in the mesial temporal lobe anterior to the hippocampus. The medial entorhinal cortex receives highly processed spatial information from a broad swath of the neocortex, primarily from somatosensory association cortex and perirhinal cortex but with important connectivity to the medial prefrontal cortex and the amygdala. It is the main gateway of neocortical information entering the hippocampus [13]–[15]. ‘Grid cells’ located in the medial entorhinal cortex whose activity is highly correlated with spatial location, speed, and direction of heading are specialized cells that exhibit high directional selectivity and are primed to encode information about the current context of movement [16]. Grid cell maps are not rigid but are quickly modified by environmental context [17], [18].

Experiments done in humans playing virtual reality video games while undergoing fMRI scanning have revealed entorhinal cortex activity modulated by perceived speed and direction of movement [19]. Activity in the left hippocampal formation specifically activates when egocentric information is preferred for spatial navigation, whereas the right hippocampal formation favors an allocentric strategy [20]. Thus, the finding of the left entorhinal cortex activity in MdDS, a disorder of chronic self-motion perception, may relate to a network that is specialized for processing spatial information using an egocentric spatial reference and which normally functions to encode direction and speed specific information.

A distinguishing feature of MdDS is the reduction in the rocking perception with re-exposure to passive motion, a unique and unexplained feature among balance disorders [4]. In essence, the external motion appears to null the motion perception. A theory for this has only been proposed as an adaptation to the prior motion stimulus. A more specific theory proposed here relates to a key function of the entorhinal cortex as a major hub of generating network oscillations, specifically theta oscillations that drive the creation of place fields within the hippocampus, the key to spatial navigation [21]–[23]. Direct stimulation of the human entorhinal cortex during epilepsy surgery improves spatial navigation skills with positive learning trials being associated with a resetting of the phase of the theta rhythm in the hippocampus [24]. One theory for the nulling effect of re-exposure to passive motion in MdDS (such as returning to sea or driving a car) is that the frequency and amplitude of the incoming vestibular and somatosensory signals override or phase cancel out an underlying oscillating rhythm.

Of relevance to MdDS is that the entorhinal cortex is one of two brain areas that can generate sustained activity in the absence of continuous input; the other being the prefrontal cortex to which it is highly connected [25]–[27]. Single pulse stimulation of entorhinal cortex can generate a recurrent loop of activity within the entorhinal cortex itself, which then spreads into the hippocampus when past threshold [28]. Periodic stimulation can also drive baseline entorhinal cortex faster or slower, depending on the period of the stimulation. One elegant study on *in vitro* brain slices showed that depolarizing electrical stimulation to the entorhinal cortex given in four second pulses raises its baseline firing rate, whereas hyperpolarizing pulses given in six second pulses gradually reduces the baseline firing rate to the point of stopping the activity [29]. Thus, baseline entorhinal cortex activity can potentially be modified by a change in the periodicity of its neuronal inputs making it is a plausible substrate for entrainment by external stimuli.

The amygdala activity seen in these data is consistent with the dependence of the entorhinal cortex on amygdala activity for efficient information transfer into the hippocampus [30]. Transfer of sensory information into the entorhinal cortex largely comes through the perirhinal cortex but studies in rat brain slices have shown that individual stimulation of perirhinal cortex is inefficient in spreading activity into the entorhinal cortex. However, concurrent stimulation of the amygdala promotes spread of activity from perirhinal cortex to the entorhinal cortex and subsequently into the hippocampus, a phenomenon that may be the basis for the role of motivational and emotional variables in memory consolidation [31]

The role of the prefrontal cortex in the generation of MdDS may be due to its strong functional and anatomical connections to both the entorhinal cortex and amygdala, specifically being shown in animal models to be inhibited by the entorhinal cortex while exerting regulatory control over both these areas [26], [32]. The reduced prefrontal connectivity seen in these data, particularly in the setting of increased connectivity with motion processing areas in posterior parietal, temporal, and occipital areas may together contribute to the increased activity with the entorhinal cortex and amygdala and contribute to a poorly regulated network where there is enhanced transmission of motion information into limbic areas with less regulatory control. Only connectivity between the EC/AG and other brain regions were investigated in this study. It is possible, however that connectivity between these areas (e.g. prefrontal and posterior parietal cortex) is also important in the generation of MdDS symptoms.

One phenomenon in MdDS that suggests a very strong role for structures involved in memory consolidation is that many patients experience a spontaneous re-emergence of their rocking dizziness even years after their original MdDS subsides [4]. The role of the amygdala may be relevant to why some patients who have an initial episode of MdDS that resolves tend to have spontaneous recurrences of the rocking sensation during periods of stress [7]. In addition, when patients experience recurrent episodes of MdDS, the episodes tend to become longer [4]. This suggests that functional connections that are created during the original period of habituation to background sea or air conditions can be strengthened with time and may develop a lower threshold for activation with subsequent stimulation.

Because of the inherent confound of concurrent mood and anxiety disorders which complicate chronic illness, we found it necessary to take extra steps to be sure that differentially active brain areas in MdDS were not simply due to anxiety or depression. The HADS is a well-validated screening tool of seven items of anxiety and seven items of depression [33]. We used scores on the HADS anxiety and depression subcomponents as nuisance covariates to remove signal that varied with high HADS subscores. However, in order to determine whether the peak voxels in the contrast images may have occurred even *near* regions implicated in anxiety and depression and may have been considered distinct simply because of thresholding, we used a multivariate regression analysis to identify brain areas that were specifically associated with high anxiety and depression scores. Using this analysis, classical brain areas that have been previously reported to be under/overactive in anxiety and depression were revealed but did not overlap with any areas seen in the primary analysis. These included the subgenual anterior cingulate cortex activity that correlated with depression [34]–[36] and dorsal midbrain and anterior temporal lobe activity correlating with anxiety [37]–[39]. There are no resting state FDG PET studies in anxiety disorders that have shown abnormalities in baseline metabolic activity in the entorhinal cortex or amygdala but amygdala activity is inducible in many anxiety subtypes when measured with fMRI [40], [41]. Baseline glucose metabolism has been found to be higher in the left amygdala in depressed patients when analysis is limited to a region of interest over the amygdala [42]. Although it is possible that the amygdala activity in our data is related to baseline depression in MdDS subjects, the entorhinal cortex activity is not explained by previous imaging findings in mood and anxiety disorders.

Because it was still possible that the findings of enhanced entorhinal cortex and amygdala activity were related to higher baseline mood symptoms that were not fully measured with the HADS questionnaire, we also undertook a functional connectivity analysis to determine whether there were different patterns in connectivity that could relate to motion processing pathways. These connectivity differences appeared to be specific to the posterior parietal and occipital areas that process visuospatial information, with connectivity being enhanced in subjects with MdDS despite overall connectivity patterns being reduced between frontal and temporal areas.

The data presented here expand the relevance of the metabolic activity and connectivity of limbic structures to a human disorder of continuous perception of self-motion. Despite MdDS being considered a rare disorder, the study of a condition where the initial adaptation to background oscillating conditions do not successfully readapt back to stable conditions would still reflect a pathway relevant to motion adaptation. These pathways would be easier to measure in MdDS patients because the symptoms are stable and persistent, unlike the transient feelings of rocking felt by most individuals after travel that can dissipate within minutes. Although the controls were matched as well as possible given age, sex, and handedness, a more ideal control group would have been one that was also matched for the motion exposure. From a practical standpoint, this was not possible since there are infinite combinations of sea, air, and land conditions that may have been relevant to why the MdDS subjects had developed their symptoms.

The question that cannot be directly answered from this study is whether the entorhinal cortex or amygdala activity is the actual *source* of the motion related activity or is only relevant to the pathways that mediate *perception*. This can only be answered with a tool with better temporal resolution where the perception of rocking can be turned on-and-off. Enhanced activity in these limbic areas, which are important drivers of brain oscillations, may be affecting downstream areas that are responsible for the actual motion perception. Areas that project to or from the entorhinal cortex and amygdala may thus be possible targets for future studies that attempt to delineate the boundary between the source of the motion relevant activity versus areas that simply allow that activity to reach consciousness. Perhaps more relevant to patients with MdDS who remain without a cure for their symptoms, is that these connectivity changes may be used as the basis for developing neuromodulation strategies that aim to treat this persistent motion disorder.

## Supporting Information

Figure S1 **Top five clusters with the highest correlation with depression scores.** Multiple regression analysis using the depression subscore of HADS showing the top five clusters with the highest correlation with depression scores. a) Right superior parietal lobule; b) Left middle temporal gyrus; c) Left inferior frontal gyrus; d) Right superior parietal lobule; e) Right anterior cingulate cortex. Images are shown at z>3.3, extent voxels: 0.
(TIF)

Figure S2 **Top five clusters with the highest correlation with anxiety scores.** Multiple regression analysis using the anxiety subscore of HADS showing the top five clusters with the highest correlation with anxiety scores. a) Right inferior temporal gyrus; b) Left postcentral gyrus; c) Left putamen; d) Left dorsal midbrain/thalamus; e) Right cuneus. Images are shown at z>3.3, extent voxels: 0.
(TIF)

Table S1 Multiple regression analysis using HADS depression and anxiety subscores showing the top five clusters with the highest correlated activity with each subscore. ke values are for clusters thresholded at z>2.56.
(DOCX)
