# Supplementary figures and images for: Metabolic and Functional Connectivity Changes in Mal de Debarquement Syndrome
Source: PLoS One. 2012 Nov 29;7(11):e49560. doi: 10.1371/journal.pone.0049560 (PMC3510214; doi:10.1371/journal.pone.0049560)

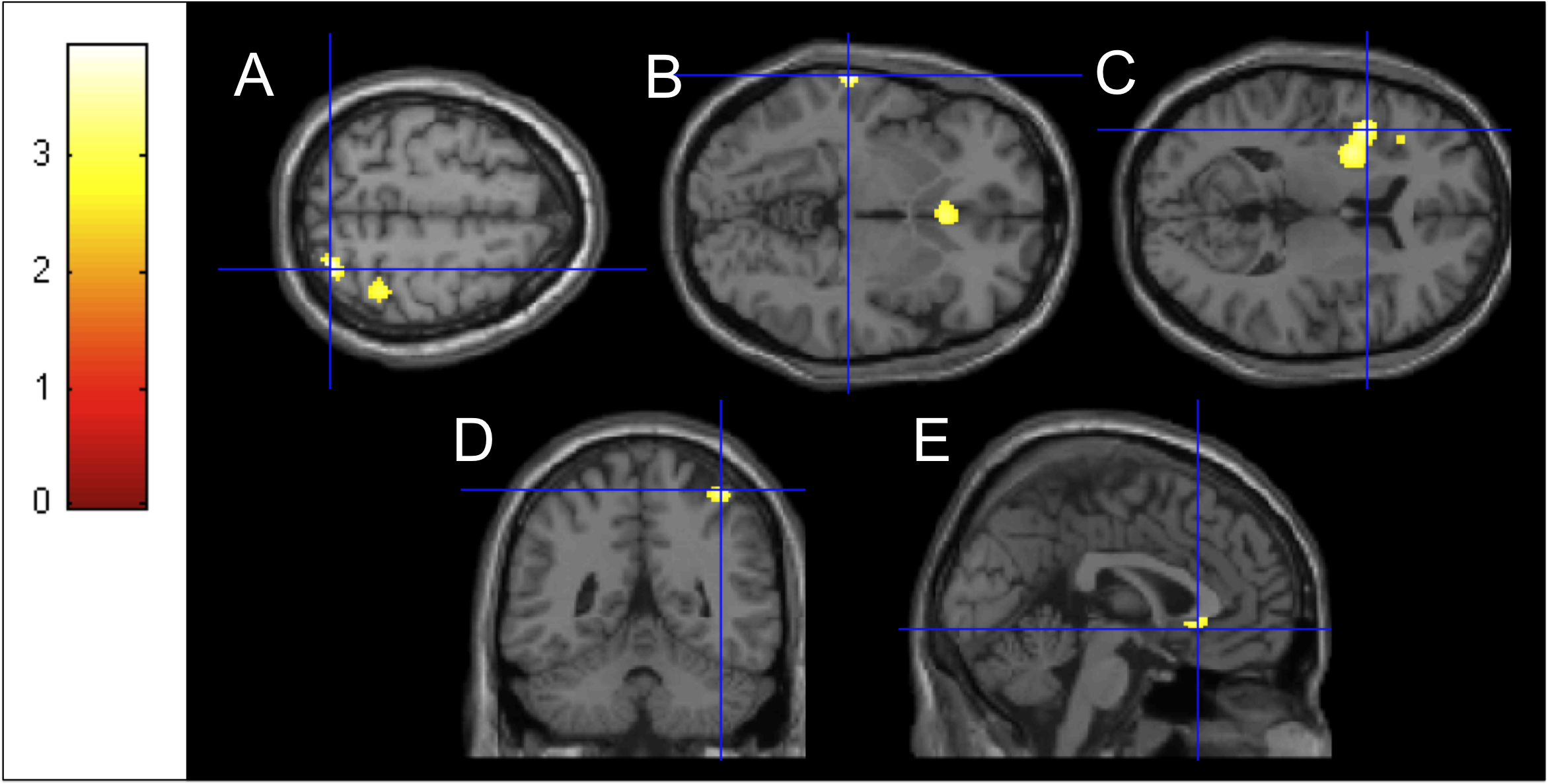

Supplement: Figure S1 — Top five clusters with the highest correlation with depression scores. Multiple regression analysis using the depression subscore of HADS showing the top five clusters with the highest correlation with depression scores. a) Right superior parietal lobule; b) Left middle temporal gyrus; c) Left inferior frontal gyrus; d) Right superior parietal lobule; e) Right anterior cingulate cortex. Images are shown at z>3.3, extent voxels: 0. (TIF) [file pone.0049560.s001.tif]

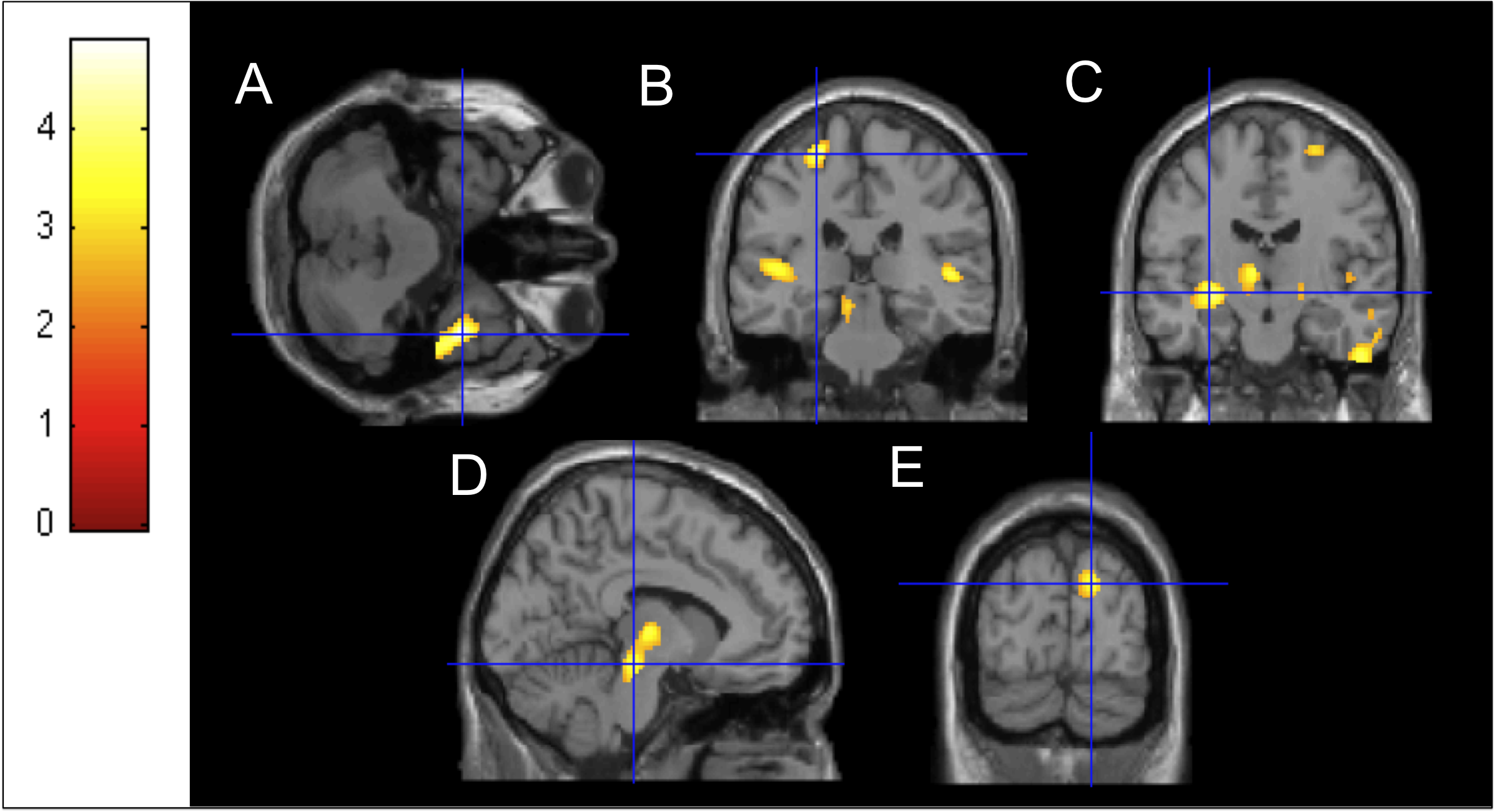

Supplement: Figure S2 — Top five clusters with the highest correlation with anxiety scores. Multiple regression analysis using the anxiety subscore of HADS showing the top five clusters with the highest correlation with anxiety scores. a) Right inferior temporal gyrus; b) Left postcentral gyrus; c) Left putamen; d) Left dorsal midbrain/thalamus; e) Right cuneus. Images are shown at z>3.3, extent voxels: 0. (TIF) [file pone.0049560.s002.tif]
