# Supplementary material for: Metabolic and Functional Connectivity Changes in Mal de Debarquement Syndrome
Source: PLoS One. 2012 Nov 29;7(11):e49560. doi: 10.1371/journal.pone.0049560 (PMC3510214; doi:10.1371/journal.pone.0049560)
Supplement: Table S1 — Multiple regression analysis using HADS depression and anxiety subscores showing the top five clusters with the highest correlated activity with each subscore. ke values are for clusters thresholded at z>2.56. (DOCX) [file pone.0049560.s003.docx]

**Table S1:** Multiple regression analysis using HADS depression and anxiety subscores showing the top five clusters with the highest correlated activity with each subscore. ke values are for clusters thresholded at z>2.56.

| **Depression** | | | | | | |
| --- | --- | --- | --- | --- | --- | --- |
|  | MNI coordinates | |  | | | |
|  | **X** | **Y** | **Z** | **ke** | ***z*** | **Location** |
| A | 28 | -70 | 58 | 226 | 3.58 | Right superior parietal lobule |
| B | -68 | -26 | -4 | 39 | 3.50 | Left middle temporal gyrus |
| C | -40 | 10 | 10 | 330 | 3.33 | Left inferior frontal gyrus |
| D | 40 | -48 | 62 | 106 | 3.12 | Right superior parietal lobule |
| E | 2 | 22 | -4 | 50 | 2.95 | Right anterior cingulate cortex |
|  | | | | | | |
| **Anxiety** | | | | | | |
|  | MNI coordinates | |  | | | |
|  | **X** | **Y** | **Z** | **ke** | ***z*** | **Location** |
| F | 44 | 0 | -40 | 621 | 4.27 | Right inferior temporal gyrus |
| G | -24 | -28 | 64 | 150 | 3.74 | Left postcentral gyrus |
| H | -30 | -14 | -6 | 1256 | 3.65 | Left putamen |
| I | -8 | -24 | -12 | 331 | 3.45 | Left dorsal midbrain/thalamus |
| J | 16 | -84 | 30 | 272 | 3.36 | Right cuneus |
